# Carbon dioxide increases with face masks but remains below short-term NIOSH limits

**DOI:** 10.1186/s12879-021-06056-0

**Published:** 2021-04-16

**Authors:** Michelle S. M. Rhee, Carin D. Lindquist, Matthew T. Silvestrini, Amanda C. Chan, Jonathan J. Y. Ong, Vijay K. Sharma

**Affiliations:** 1 Theranova LLC, 101 Mississippi Street, San Francisco, CA 94107 USA; 2 Division of Neurology, National University Hospital and Yong Loo Lin School of Medicine, National University of Singapore, NUHS, Tower Block, 1E Kent Ridge Road, 119228 Singapore City, Singapore

**Keywords:** Personal protective equipment (PPE), N95, Face mask, Respirator, Powered air purifying respirator (PAPR), Carbon dioxide (CO2)

## Abstract

**Background and purpose:**

COVID-19 pandemic led to wide-spread use of face-masks, respirators and other personal protective equipment (PPE) by healthcare workers. Various symptoms attributed to the use of PPE are believed to be, at least in part, due to elevated carbon-dioxide (CO2) levels. We evaluated concentrations of CO2 under various PPE.

**Methods:**

In a prospective observational study on healthy volunteers, CO2 levels were measured during regular breathing while donning 1) no mask, 2) JustAir® powered air purifying respirator (PAPR), 3) KN95 respirator, and 4) valved-respirator. Serial CO2 measurements were taken with a nasal canula at a frequency of 1-Hz for 15-min for each PPE configuration to evaluate whether National Institute for Occupational Safety and Health (NIOSH) limits were breached.

**Results:**

The study included 11 healthy volunteers, median age 32 years (range 16–54) and 6 (55%) men. Percent mean (SD) changes in CO2 values for no mask, JustAir® PAPR, KN95 respirator and valve respirator were 0.26 (0.12), 0.59 (0.097), 2.6 (0.14) and 2.4 (0.59), respectively. Use of face masks (KN95 and valved-respirator) resulted in significant increases in CO2 concentrations, which exceeded the 8-h NIOSH exposure threshold limit value-weighted average (TLV-TWA). However, the increases in CO2 concentrations did not breach short-term (15-min) limits. Importantly, these levels were considerably lower than the long-term (8-h) NIOSH limits during donning JustAir® PAPR. There was a statistically significant difference between all pairs (*p* < 0.0001, except KN95 and valved-respirator (*p* = 0.25). However, whether increase in CO2 levels are clinically significant remains debatable.

**Conclusion:**

Although, significant increase in CO2 concentrations are noted with routinely used face-masks, the levels still remain within the NIOSH limits for short-term use. Therefore, there should not be a concern in their regular day-to-day use for healthcare providers. The clinical implications of elevated CO2 levels with long-term use of face masks needs further studies. Use of PAPR prevents relative hypercapnoea. However, whether PAPR should be advocated for healthcare workers requiring PPE for extended hours needs to evaluated in further studies.

## Introduction

Wide-spread use of face-masks has been brought on by the current COVID-19 pandemic. Public health officials have recommended face-masks since studies demonstrated that they reduce SARS-CoV-2 transmission [1, 2]. However, this recommendation became controversial and even politicized in some countries, because of concerns about the safety of masks [3, 4]. Furthermore, some studies raised concerns related to the hypercapnoea and hypoxemia caused during donning the face masks [4–7].

The personal protective equipment (PPE) include N95 respirators, valved-respirators and powered air purifying respirator (PAPR), in addition to face shields and goggles. Although PPE use is necessary, the side-effects become more noticeable with their prolonged use [8, 9]. These perceptions have been associated not only with mask fit but also with carbon dioxide (CO2) rebreathing from the mask [10–12]. Numerous side-effects such as dyspnea, dizziness, reduced cognition and headaches, have been reported with mask use, particularly with the tight-fitting N95 masks and valved respirators [4, 8, 9].

CO2 is a colorless, odorless gas that is a natural by-product of respiration. In normal room-air, CO2 levels are around 0.03–0.04%, equivalent to 300–400 ppm (ppm) and have no known toxic effect [13, 14]. However, studies have shown that short-term exposure to CO2 levels above 1000 ppm start affecting cognitive function and at much higher levels, can be toxic to the human body [15–18]. The National Institute for Occupational Safety and Health (NIOSH) has an 8-h threshold limit value - time-weighted average recommended exposure limit (TLV-REL) of 5000 ppm and a 15-min threshold limit value - short term exposure limit (TLV-STEL) of 30,000 ppm for CO2 in workplace ambient air [13].

It is known that dead space (respirator volume) and hypoventilation related to breathing resistance in respirators can contribute to CO2 rebreathing, thereby increasing the CO2 to symptomatic levels [11, 12, 19, 20]. We evaluated the local concentration of CO2 under various PPE to determine its potential role as a causative factor in the symptomatology described with prolonged use [21]

## Methods

### Study population

A total of 11 volunteers were enrolled in the study. Inclusion criteria for the study were: participants had to be at least 18 years old, and capable and willing to follow all study-related procedures. All participants underwent a thorough physical examination, especially of cardiovascular and respiratory system. Participants with chronic obstructive pulmonary disease, respiratory failure, active chest infection, ischemic heart disease and cardiac failure were excluded. Additionally, we excluded participants with baseline pulse-oximeter saturation below 95% while breathing ambient air. This was a single-site study and the experimental session for each individual took place over a single 2-h session for CO2 measurements. The study was approved by the research ethics committee of Theranova, LLC, US.

### Study materials

**Fig. 1:**
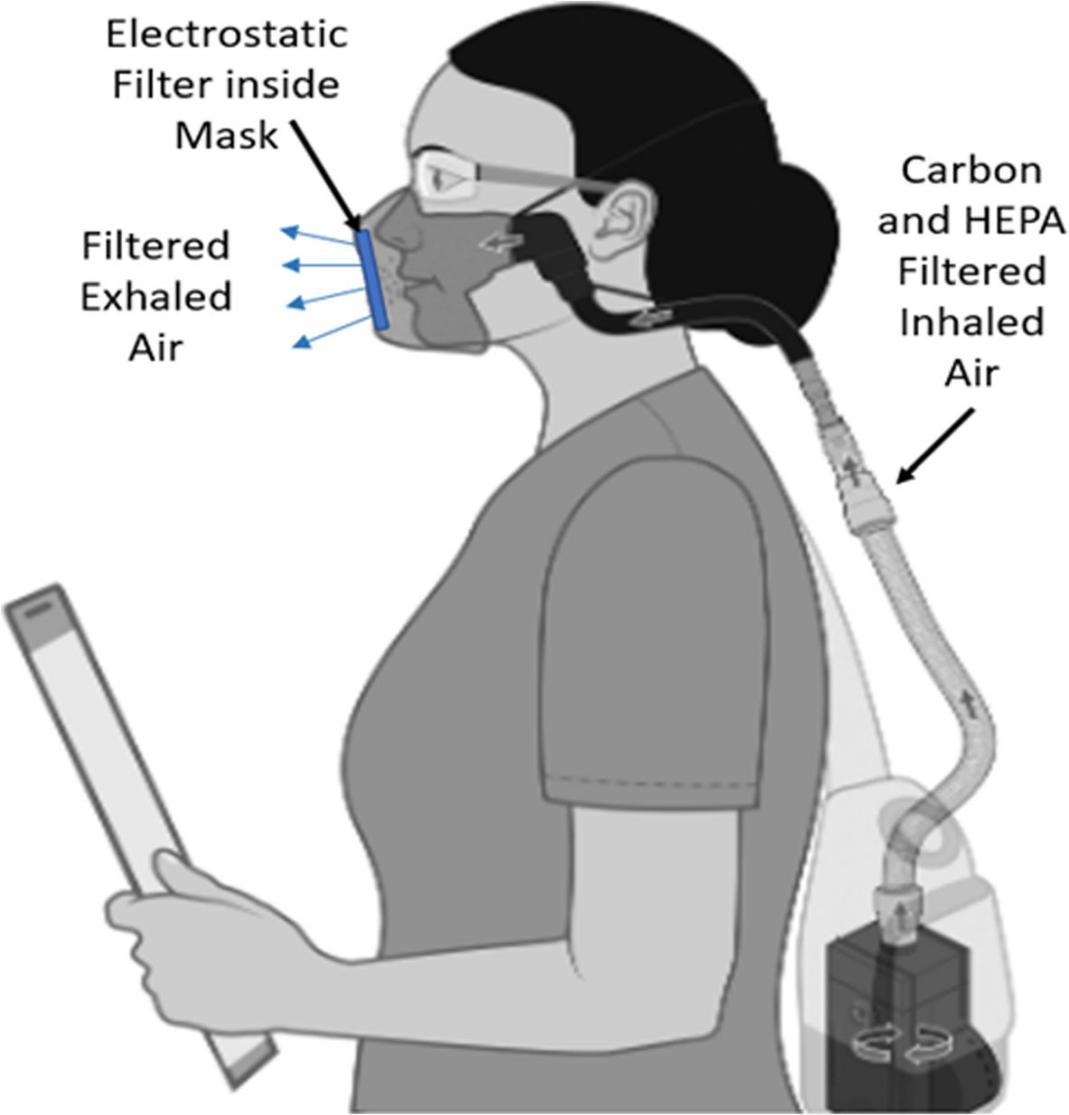
JustAir powered air purifying respirator (PAPR) donned by a cartoon showing the 2 filters that protect the user from contracting as well as transmitting the viral infection

All PPE devices were commercially available. The KN95 respirator (Emercate, ShenZen, China) and valved-respirator model 7502/37082(AAD) (3 M, St. Paul, MN) were chosen due to their ubiquity in healthcare practice. The PAPR (JustAir®, San Francisco, CA) was chosen due to its dual filtration system that included high-efficiency particulate air (HEPA) filtration of air delivered (constant airflow of 150L per minute) to the mask and electrostatic filtration of air leaving the mask (Fig. 1). The latter filtration is critical in order to prevent the potential spread of infectious agents from an asymptomatic individual since the JustAir® PAPR generates high volume of air.

### Data collection

We used the commercially available GASLAB Model CM-0123 ExplorIR-W 20% CO2 Sensor Development Kit. The ExplorIR®-W is a low power, high performance CO2 sensor that can measure up to 20% CO2 levels with an accuracy of ±70 ppm / ± 5% of reading. It is suitable for measuring high concentrations of CO2 in closed-loop sampling applications on battery operation in portable sampling instruments.

The CO2 sensor was attached to a thin nasal cannula that was worn by the study participants to measure CO2 levels directly at the nasolabial fold. Soft paddings of JustAir® PAPR prevented any air leak due to placement of nasal canula. Although, the participants underwent mask-fitting test for KN95 and valved-respirator, we feel that there could have been minor air leak with all 3 types of PPE. Since our aim was to evaluate the relative changes in CO2 levels with each PPE, such minor leaks would not have affected our finding. Patients were asked to sit at rest while CO2 measurements were taken for each group in four consecutive 15-min intervals. In the first 15-min test, no mask was used, which served as baseline. The next 3 tests were for the different configurations- KN95 respirator, valved-respirator and JustAir® PAPR. The order of the mask testing was randomized for each participant. The sensor module was attached to a computer, and GasLab® software was used to measure and record CO2 at 1-Hz sampling rate.

Peripheral oxygen saturation (SpO2) and heart rate (HR) were also measured continuously with a Rad-8 bedside pulse oximeter (Masimo, Irvine, CA) during each 15-min session.

All study methods were carried out in accordance with institutional guidelines and regulations.

### Statistical analysis

CO2 levels were expressed in parts per million (ppm) and percentages (%). The effects of no mask, PAPR, KN95, and valved-respirator exposure on CO2 levels were analyzed with the use of repeated measures analysis of variance (ANOVA) with multiple comparisons with post-hoc Tukey test. A *p*-value of < 0.05 was considered as statistically significant. Statistical analyses were performed using the Prism statistical program version 9.0.0 for Windows and Mac (Graphpad Software, 1989, San Diego, CA, USA).

## Results

### Patient population

Eleven volunteers were enrolled in the study. Median age of the study participants was 32 years (range 18–54) and consisted 6 (55%) men. All study participants provided written informed consent.

### Oxygen saturation (SpO2) level and heart rate

**Fig. 2:**
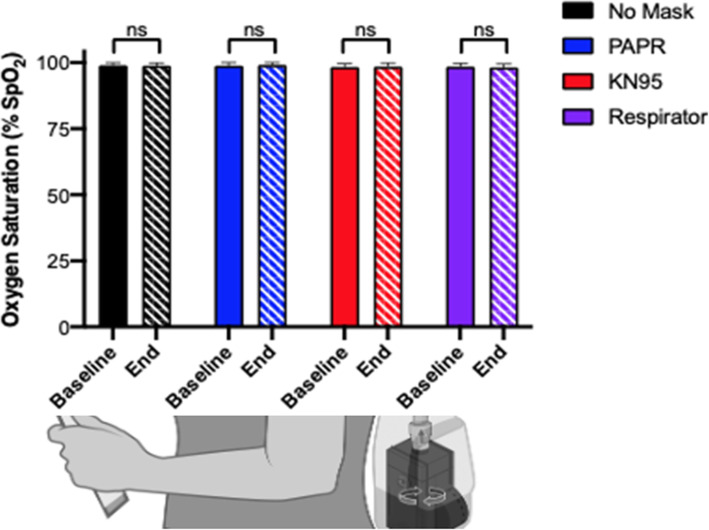
Mean SpO2 at baseline and endpoint for each study interval. No significant changes were seen between study groups and within individuals

**Fig. 3:**
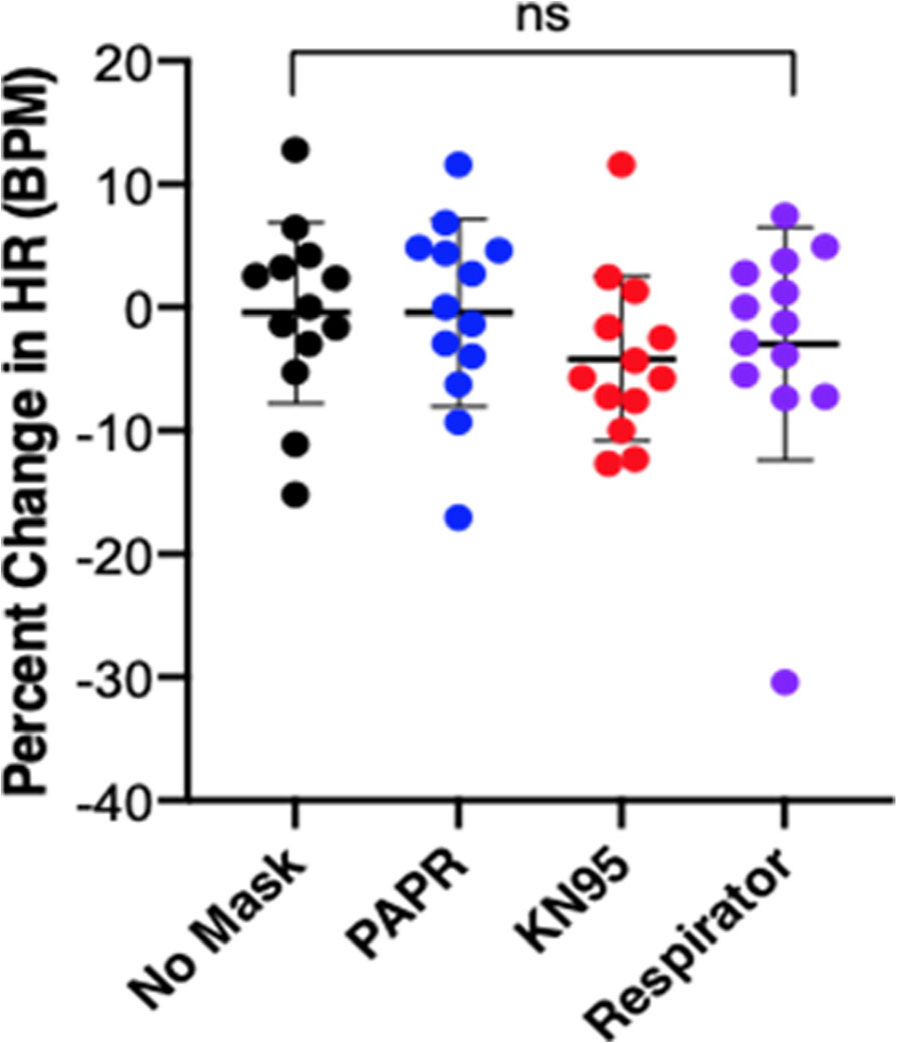
Percent change in HR for each study interval. No significant changes were seen between study groups and within individuals

Mean SpO2 at baseline were 99% for no mask, 98% for JustAir® PAPR, 98% for KN95 respirator, and 98% while donning the valved-respirator (Fig. 2). Percent mean (SD) changes in hear rate were − 0.44 (7.3) for no mask, − 0.44 (7.6) for JustAir® PAPR, − 4.1 (6.7) for KN95 respirator and − 3.0 (9.5) for valved-respirator (Fig. 3). These levels did not change significantly for each individual and between the study groups throughout the study.

### CO2 levels

**Fig. 4:**
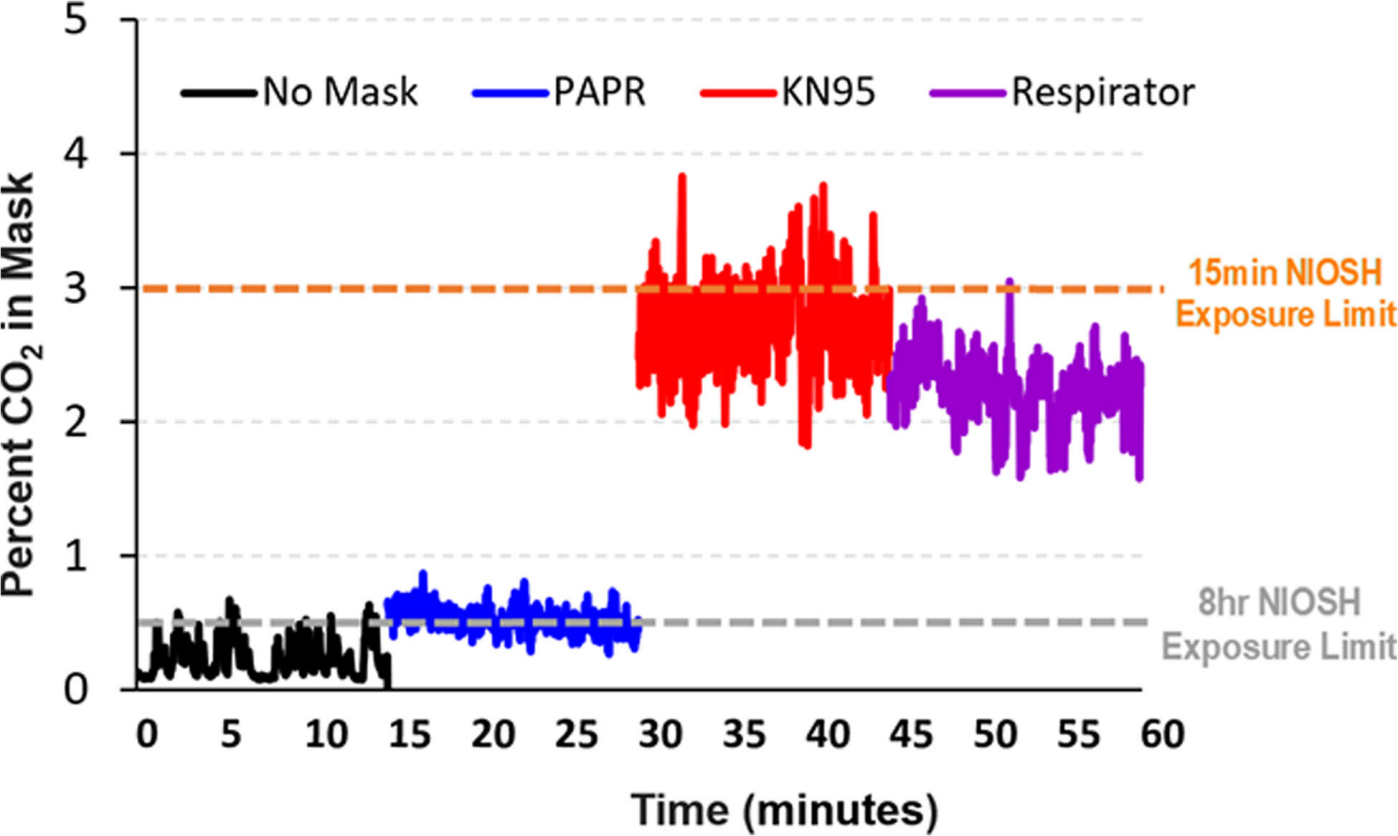
Continuous CO2 levels over time for 1 individual with 15 min of data for each group. NIOSH exposure limit thresholds added for reference

**Fig. 5:**
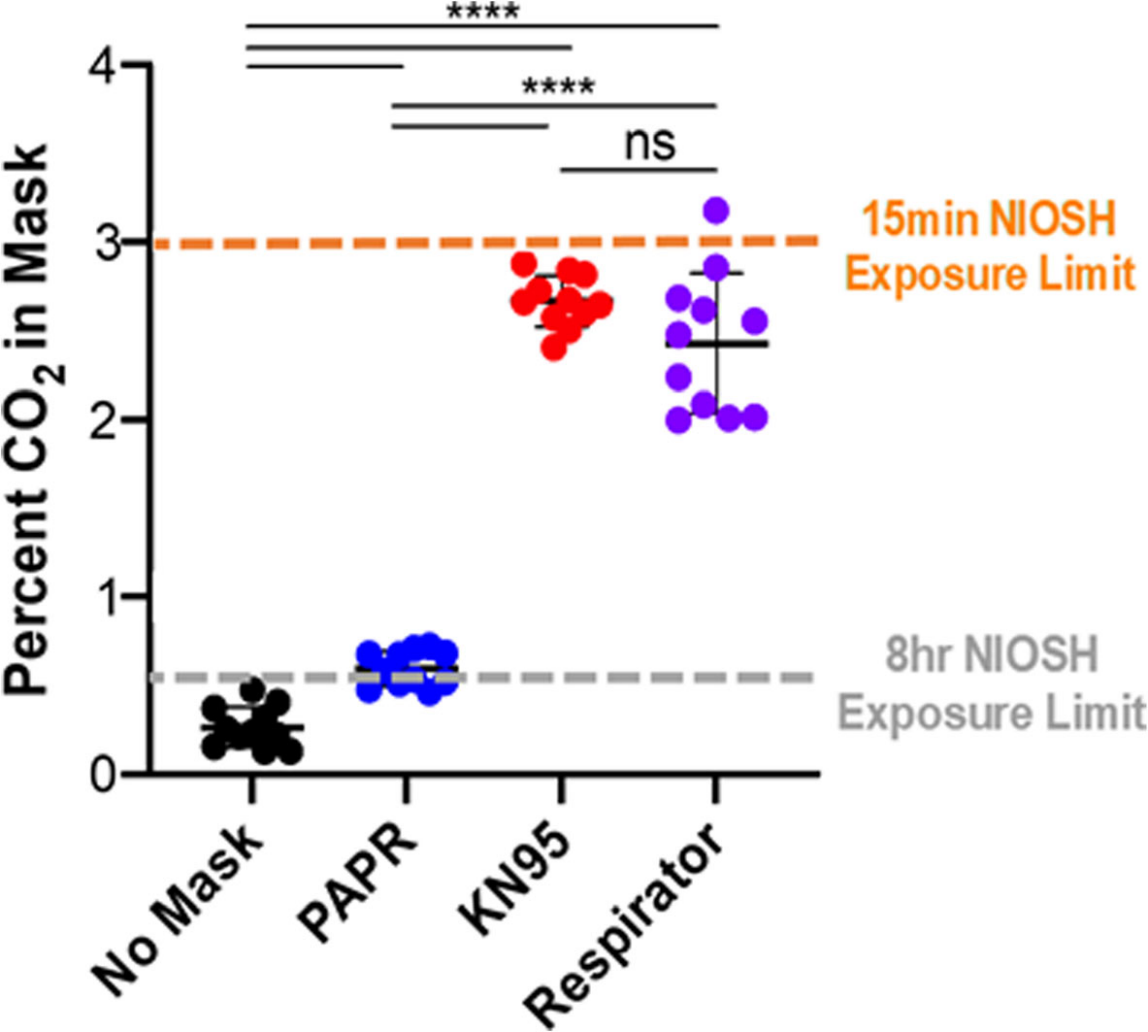
Average % CO2 levels between no mask, KN95, respirator and JustAir. All differences between each group were significant except between KN95 and respirator (**** *p* < 0.0001). Eight hour and 15 min NIOSH recommended levels of exposure depicted

Fig. 4 depicts the serial changes in CO2 levels over time for one individual. Overall, the mean CO2 with no mask was 0.27% when breathing ambient air with a CO2 concentration of 0.04%. The percent mean (SD) CO2 values for no mask, JustAir® PAPR, KN95 respirator, and valved-respirator were 0.26 (0.12), 0.59 (0.097), 2.6 (0.14) and 2.4 (0.59), respectively with the NIOSH levels depicted as reference (Fig. 5). The 2.4–2.6% CO2 concentration translates into a 10-fold increase in CO2 with KN95 respirator and valved-respirator or 24,000–26,000 PPM at the nasolabial fold, which is greater than the NIOSH 8-h TLV-REL of 5000 PPM. Although, there was approximately a 4-fold reduction of CO2 with PAPR to 0.59% or 5900 ppm, it still remained slightly greater than the NIOSH 8-h TLV-REL of 5000 PPM. Overall, use of respirators resulted in significant increases in CO2 concentrations, which exceeded the 8-h NIOSH exposure threshold limit for TWA-REL. However, the increases in CO2 concentrations did not breach short-term (15-min) limits. Importantly, these levels were considerably lower than the long-term (8-h) NIOSH limits during donning JustAir® PAPR.

The repeated measures ANOVA for CO2 yielded a statistically significant overall effect of KN95 and valve respirator (*p* < 0.0001). The Tukey post-hoc multiple comparisons revealed statistically significant differences in mean CO2 concentration between the following pairs: no mask vs. KN95 (*p* < 0.0001), no mask vs. respirator (*p* < 0.0001), no mask versus JustAir® PAPR, JustAir® PAPR versus KN95 respirator (*p* < 0.0001), JustAir® PAPR versus valved-respirator (*p* < 0.0001). The difference between KN95 and valve respirator was not significant (*p* = 0.25).

## Discussion

Our study demonstrates that there was an elevated concentration of CO2 in range that is in excess of NIOSH limits under passive KN95 respirator and valved-respirators. The average CO2 concentrations in a 15-min time interval were greater than 20,000 ppm, but below the 15-min short-term exposure limit. However, those average concentrations were significantly above the NIOSH upper limit of 5000 ppm for 8 h TWA-REL. Although, the participants donned various PPEs only for 15-min each, we hypothesize that the CO2 concentrations reached a steady state by 15-min and should be equivalent to when used for 8 h. Interestingly, this CO2 rise was much lower while donning JustAir® PAPR and was only slightly above the safety limits determined by NIOSH. We believe that the small size of the face mask used in the JustAir PAPR might have contributed to the observed CO2 values. Perhaps a slightly bigger mask would resolve this issue*.* We did not observe any significant changes in SpO2 and heart rate regardless of respirator type, echoing the findings in other studies [5, 10].

Our findings have significant implications for health care personnel who are required to wear PPE for long periods of time. Elevated CO2 has been reported to result in hemodynamic changes in the intracranial arteries and considered a contributor towards discomfort, fatigue, dizziness, headache, shortness of breath, generalized weakness, lethargy and drowsiness [10]. Furthermore, these symptoms increased with prolonged use of the face mask [8, 19]. Some studies even showed proportional decrease in cognitive abilities with increasing CO2 levels [15–18]. At least to some extent, these symptoms have been believed to reduce the compliance to PPE use [15] and may even affect decision making capabilities during work shifts. Healthcare workers are expected to work with full efficiency and therefore deserve PPE that are comfortable, can be worn for extended periods and do not impair cognitive abilities. Symptoms related to an increase in CO2 concentration are believed to start above the NIOSH 8-h threshold of 5000 ppm [2, 17]. Our study showed that during donning JustAir® PAPR, CO2 concentration remained much lower as compared to KN95 respirator and valved-respirators use. However, the CO2 concentration was still slightly higher than the NIOSH 8-h threshold. Although difficult to substantiate, we believe that the relatively smaller size of the face mask could have contributed to this observation. Use of respirators (KN95 mask and valved-respirator) resulted in significant increases in CO2 concentrations, which exceeded the 8-h NIOSH exposure threshold limit for TWA-REL**.** However, the increases in CO2 concentrations did not breach short-term (15-min) limits.

Our findings are consistent with a recent study that reported normalization of cerebral hemodynamic parameters when another type of PAPR (3 M® Versaflo® TR-300 series) was donned on top of the N95 respirator [3]. Although we did not evaluate whether PAPR reduces mask related headache and other symptoms, we believe that the positive pressure generated by the PAPR results in lower CO2 level by positive pressure-assisted exhalation, in addition to maintaining the O2 concentration inside the PAPR, thereby providing an improvement in headaches and other side-effects related to the use of respirators [10]

We acknowledge certain limitations of our study. First, the study has a small sample size. However, this is only an observational “proof of concept” study to evaluate the serial changes in end-tidal CO2 with various PPE. Furthermore, our observations were consistent in all the study participants. Second, we monitored the end-tidal CO2 levels when the participants were sitting. Since healthcare workers are often physically quite active while donning the PPE, it is difficult to comment whether CO2 responses would have been similar if we had performed ambulatory monitoring. Although, the size and weight of the monitoring equipment would make such study difficult, we hypothesize that CO2 levels would be even higher if a similar study could have been performed during a real work-shift when participants were exerting themselves as well as talking [12]. Third, we did not test the clinical side-effects (especially headache) of increased CO2 level among the users of N95 respirators. We wish to reiterate that the primary aim of this pilot study was to evaluate the short-term changes on CO2 levels. Lastly, instead of performing large scale complex studies, we felt the need to expedite these results, given that the pandemic is worsening in certain countries.

## Conclusions

Our study demonstrates a significant increase in end-tidal CO2 concentrations among healthy volunteers while donning KN95 respirator, valved-respirator as well as PAPR. However, the CO2 rise during donning PAPR was consistently lower when compared to the K95 and valved-respirator. Therefore, there should not be a concern in their regular day-to-day use for healthcare providers. The clinical implications of elevated CO2 levels with long-term use of passive masks needs further studies. Use of PAPR prevents relative hypercapnoea. We recommend further studies to evaluate whether PAPR (like JustAir® alone which provides adequate filtration of viral particles both during inhalation and exhalation) should be advocated for healthcare workers requiring PPE for extended hours. Also, further research is needed to determine if PAPR is more comfortable and, reduce symptoms such as headaches and does not impair cognitive performance.

## Data Availability

The datasets used and/or analysed during the current study are available from the corresponding author on reasonable request.
